# Composite fiber Bragg grating written by femtosecond laser for Raman suppression in high-power fiber oscillators

**DOI:** 10.1007/s12200-025-00165-3

**Published:** 2025-10-21

**Authors:** Hao Li, Rong Zhao, Binyu Rao, Xinyu Ye, Baolai Yang, Meng Wang, Zhixian Li, Zilun Chen, Zefeng Wang, Jinbao Chen

**Affiliations:** 1 College of Advanced Interdisciplinary Studies, National University of Defense Technology, Changsha, 410073 China; 2 Nanhu Laser Laboratory, National University of Defense Technology, Changsha, 410073 China; 3 State Key Laboratory of Pulsed Power Laser Technology, Changsha, 410073 China

**Keywords:** Fiber Bragg grating, Fiber laser, Stimulated Raman scattering, High-power laser, Femtosecond laser

## Abstract

High-power fiber oscillators have been widely used in industrial processing, high-end manufacturing, biomedicine and so on. However, as the output power increase, stimulated Raman scattering (SRS) becomes the main factor limiting the performance improvement of fiber oscillators. In this paper, a chirped and tilted fiber Bragg grating (CTFBG) is used to suppress SRS in a high-power fiber oscillator. The CTFBG is fabricated on one side of a low-reflectivity FBG (LRFBG) to form a composite FBG by the femtosecond laser phase mask technology, enhancing the compactness and stability of the fiber oscillator system. SRS is effectively suppressed by CTFBG with a Raman suppression depth and width of 16 dB and 86 nm, respectively, and the Raman light ratio in the output power decreases by an order of magnitude. The output power of fiber oscillators is increased to 9 kW, which is the highest power for fiber oscillators with SRS suppression using CTFBGs, to the best of our knowledge. This work demonstrates that the composite FBG can effectively improve the performance of high-power fiber oscillators, which provides new insights into the development of fiber laser technology.

**Graphical Abstract:**

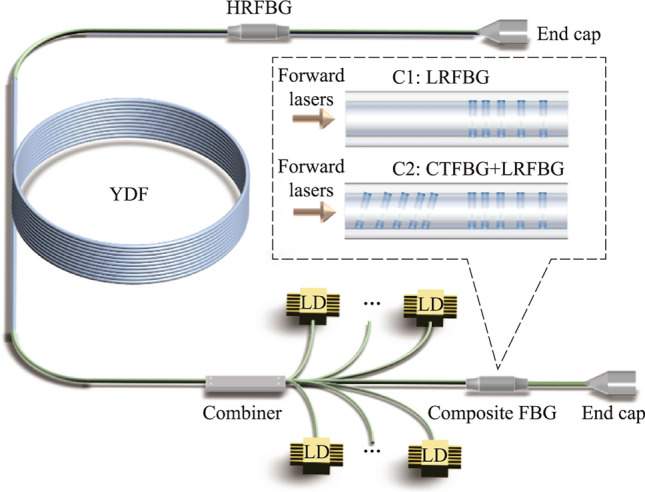

## Introduction

Due to the characteristics of high conversion efficiency, good beam quality and flexible operation [1], high-power fiber lasers (HPFLs) are increasingly used in the field of industrial processing and high-end manufacturing. Based on the structural characteristics, HPFLs can be divided into amplifiers and oscillators. Compared with amplifiers, the oscillators are more widely used in the high-end manufacturing due to their compact structure, simple control logic and strong anti-reflection ability [2–4]. To achieve efficient and precise material processing capabilities, fiber oscillators need to have high power, high efficiency and good beam quality. At the same time, the length of the transmission fiber also needs to be increased to enhance ability of flexible operation, realizing a lager processing area. Up to now, the maximum power of all-fiber oscillators has exceeded 10 kW [5]. However, as the power and fiber length increase, stimulated Raman scattering (SRS) becomes the main obstacle to improving performance of fiber oscillators [2]. SRS can cause the conversion of signal light to Raman light, reducing the output power and efficiency of the fiber laser system, which not only affects the processing quality but also increases the risk of system damage, especially in material processing. Since the material surface can reflect some Raman light back into the fiber laser system, the backward Raman light further enhances SRS [6]. To address this issue, researchers have proposed various methods to suppress SRS in the fiber laser system, such as increasing the mode field area of the fiber [7], fabricating spectrally selective fibers [8], using fiber gratings as spectral filters [9, 10]. In comparison, using fiber gratings as spectral filters to suppress SRS has the advantages of simple operation, low cost, easy compatibility with fiber systems, and the ability to filter out backward Raman light, which has led to its wider application. There are two types of fiber gratings that can be used to suppress SRS: chirped and tilted fiber Bragg gratings (CTFBGs) [10–17] and long-period fiber gratings (LPFGs) [9, 18–20]. Compared with LPFG, CTFBG is insensitive to temperature and stress changes, and has a better spectral stability, making it more suitable for suppressing SRS in fiber oscillators. Thus, there have been continuous reports of using CTFBG to suppress SRS in HPFLs in recent years [11–17], which has also promoted the commercialization of CTFBG [21–23]. Obviously, the fiber Bragg gratings (FBGs) are key fiber components in high-power fiber oscillators. On the one hand, the FBGs can be used as resonator cavity mirrors o to select wavelength and couple output power [24–26]. On the other hand, CTFBGs can act as spectral filters to suppress SRS. However, the fabrication method for mirror FBG and CTFBG is almost exclusively the traditional UV laser phase mask technology, which requires hydrogen loading and high-temperature annealing of the fiber [11]. Not only is the preparation period of the FBG long, but also the FBG is easy to generate heat during operation. This is because when the annealing is incomplete, the residual hydrogen and the hydroxyl groups generated during the inscription process in FBGs will absorb near-infrared lasers and generate heat, making it difficult to increase the power handling capability. So far, the maximum handling power of the UV-written mirror FBG and CTFBG has been reported to be 8 kW [3] and 3.4 kW [27], respectively, limiting their application in high-power fiber oscillators.

Femtosecond (fs)-lasers offer a new solution for the fabrication of FBGs [28–31]. Since fs-laser has no requirement for fiber photosensitivity, FBG can be directly inscribed in the fiber by fs-lasers and the fiber does not need to be hydrogen-loaded. This effectively overcomes the shortcomings of the UV exposure method, which not only shortens the fabrication period and cost of FBGs but also effectively solves the problem of FBG heating caused by hydrogen and hydroxyl groups. In addition, fs laser-induced refractive index changes in fibers are stable, and have the characteristic of high temperature resistance [32, 33], which is different from the refractive index changes formed by laser-induced atomic gratings; the latter are dynamically tunable periodic refractive index gratings [34]. To date, the reported maximum handling power of fs-written mirror FBG and CTFBG is 10 kW [5] and 4 kW [35], respectively. However, the handling power of the fs-written CTFBG needs to be increased to further verify the SRS suppression effect of fs-written CTFBG in high-power fiber oscillators. Furthermore, we recently reported an effective and simple strategy for SRS suppression using CTFBGs in 3.5 kW fiber oscillators by introducing the CTFBG into one side of the low-reflectivity FBG (LRFBG) within the resonant cavity [36]. However, there is a high risk of damage to the splice point between CTFBG and LRFBG, which can easily cause fiber oscillator failure.

In this paper, the CTFBG and LRFBG are inscribed on the same 30 μm/250 μm fiber to form a composite FBG using fs-laser phase mask technology, which avoids the increase in splice point caused by introducing CTFBG, improving the compactness and stability of laser system. SRS is effectively suppressed with a Raman suppression depth and width of 16 dB and 86 nm, respectively, and the maximum output power of the fiber oscillator is increased by 140 W to 9 kW, corresponding to the slope efficiency of 83.4%. To the best of our knowledge, this is the highest power for fiber oscillators with SRS suppression using CTFBGs. This work can contribute to the suppression of the SRS effect in high-power fiber oscillators, which will promote the more extensive and efficient application of HPFLs.

## FBGs fabrication and measurement

**Fig. 1:**
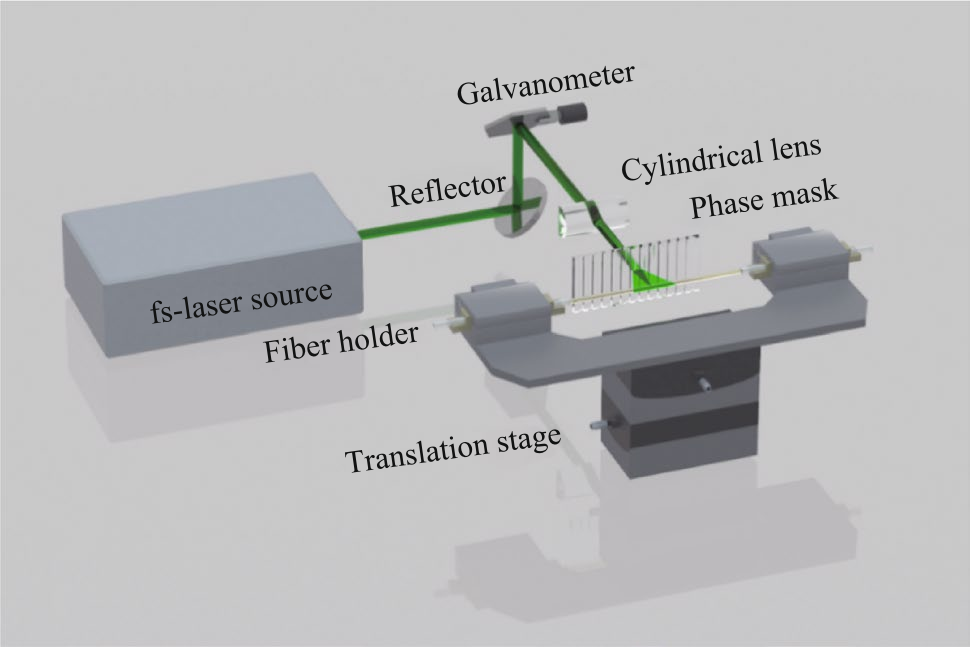
Schematic of inscription system based on fs-laser phase mask scanning technology

The mirror FBG and CTFBG are fabricated in large-mode-area double-cladding-fibers (LMA-DCF) with a core diameter of 30 μm by using a fs-laser inscription system. Figure 1 presents the inscription system based on the fs-laser phase mask scanning technology. The inscription system mainly consists of a fs-laser source (515 nm output wavelength, 290 fs pulse duration, 1 kHz repetition rate), reflector, galvanometer, cylindrical lens (25 mm focal length), phase mask (1488 nm pitch period and 2 nm/cm chirp rate for the mirror FBG; 1586 nm pitch period and 2 nm/cm chirp rate for the CTFBG), fiber holder and high-precision electric transmission stage. After the collimated fs-laser is emitted from the light source, it is reflected by the reflector and galvanometer, then vertically incident the cylindrical lens and the phase mask, and finally focused on the fiber core. In fact, the fs-laser is not only focused by the cylindrical lens but also refocused by the fiber’s circular surface. Thus, the actual waist diameter is only about 3.7 μm [37], which is much smaller than the core diameter of 30 μm. To expand the refractive modulation region, the fs-laser is needed to scan the entire fiber core. Due to the difference in grating structure, different scanning schemes are adopted to inscribe the mirror FBG and CTFBG.

**Fig. 2:**
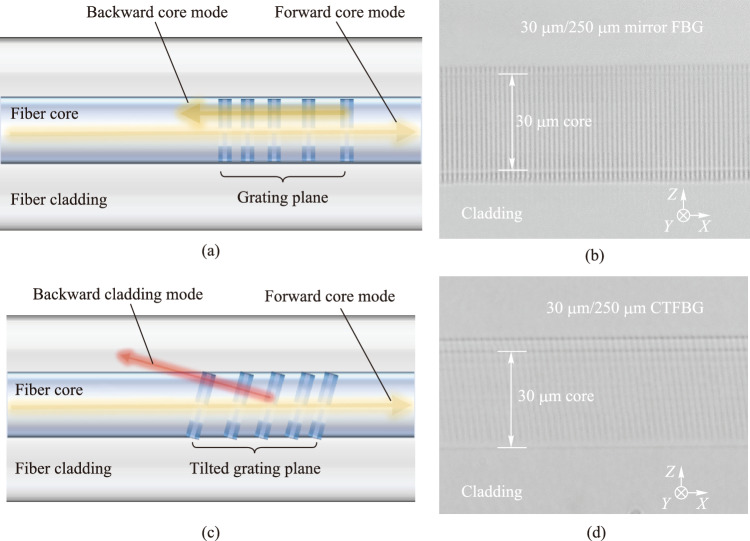
Schematic diagram of coupling characteristics and grating structure of **a** mirror FBG and **c** CTFBG. The microscope images of the side of **b** cavity mirror FBG and **d** CTFBG

Figure 2a presents the schematic diagram of coupling characteristics and grating structure of the mirror FBG. The forward core mode is coupled to the backward core mode by the mirror FBG, and the grating planes are perpendicular to the axial direction of the fiber core. Therefore, in the process of inscribing mirror FBGs, the position of the fiber is unchanged, and the fs-laser is scanned perpendicular to the axial direction of the fiber core through the vibration of the galvanometer, forming vertical grating planes. The grating structure of fs-written mirror FBG is observed by using an optical microscope, as shown in Fig. 2b. The vertical grating planes are clearly visible and completely cover the fiber core, indicating that the galvanometer-based vertical scanning scheme effectively expands the refractive index modulation region and forms a high-quality mirror FBG structure. Figure 2c presents the schematic diagram of the coupling characteristics and grating structure of the CTFBG. The forward core mode is coupled to the backward cladding mode by the CTFBG, and the grating planes are tilted to the axial direction of the fiber core. Thus, when the CTFBG is inscribed, the optical path of the fs-laser is unchanged, and the fiber is moved obliquely by the electric translation stage, so that the fs-laser scans obliquely relative to the axial direction of the fiber core, forming tilted grating planes. Figure 2d presents the microscope images of the fs-written CTFBG. The tilted grating planes are clearly visible and completely cover the fiber core, indicating that the translation-stage-based tilted scanning scheme effectively expands the refractive index modulation region and forms a high-quality CTFBG structure.

**Fig. 3:**
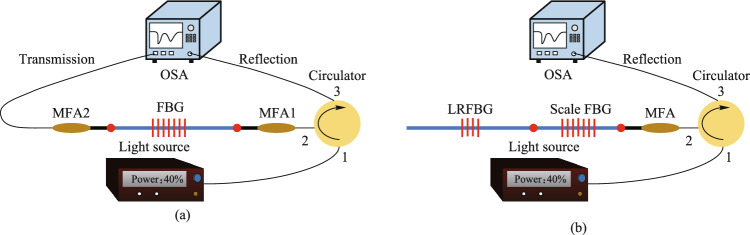
**a** Conventional multimode FBG spectrum measurement system. **b** The multimode LRFBG spectrum measurement system with scale FBG

The accurate measurement of the FBG spectrum is a significant work. Figure 3a presents the conventional multimode FBG spectrum measurement system, which is composed of the light source, fiber circulator, mode field adapter (MFA), and optical spectrum analyzer (OSA). Because the FBG is inscribed in multimode fibers, in order to avoid the influence of high-order modes excited by mode field mismatch on the accurate measurement of the fundamental mode FBG spectrum, the MFA is spliced between single-mode fiber and multimode fiber to filter out high-order modes. However, it is difficult for the conventional multimode FBG spectrum measurement system to accurately obtain the reflectivity of multimode low-reflectivity FBG (LRFBG) from the transmission spectrum. The fundamental mode reflectivity of LRFBG used as a cavity mirror is usually between 6% and 13%, and the corresponding fundamental mode transmission spectrum depth is between 0.3 dB and 0.6 dB. When measuring the transmission spectrum of multimode LRFBG (e.g., 30 μm/250 μm LRFBG), there are many high-order modes in the 30 μm/250 μm fiber, MFA cannot completely filter out the high-order modes, resulting in relatively large fluctuation of the transmission spectrum (usually greater than 1 dB), so it is difficult to accurately read the transmission spectrum depth of the fundamental mode of 30 μm/250 μm LRFBG, and the corresponding fundamental mode reflectivity cannot be obtained. To accurately measure the reflectivity of multimode LRFBG, a multimode LRFBG spectrum measurement system with scale FBG was constructed, as shown in Fig. 3b. The fiber type of the scale FBG is the same as that of the multimode LRFBG and the scale FBG is connected between the MFA and the multimode LRFBG. The other components of this FBG spectrum measurement system are the same as the traditional FBG spectrum measurement system. The reflectivity of the scale FBG is known, and its Bragg wavelength is different from that of the multimode LRFBG. Therefore, by comparing the reflection peak intensity difference between the scale FBG and the multimode LRFBG in the reflection spectrum, the reflectivity of the multimode LRFBG can be calculated as follows:

1 $${R}_{\text{L}}={R}_{\text{S}}\times {10}^{-\frac{\Delta I}{10}},$$

where *R*_L_ is the reflectivity of multimode LRFBG; *R*_S_ is the reflectivity of the scale FBG. The unit of *∆I* is dB, which is the reflection peak intensity difference between scale FBG and multimode LRFBG in the reflection spectrum.

**Fig. 4:**
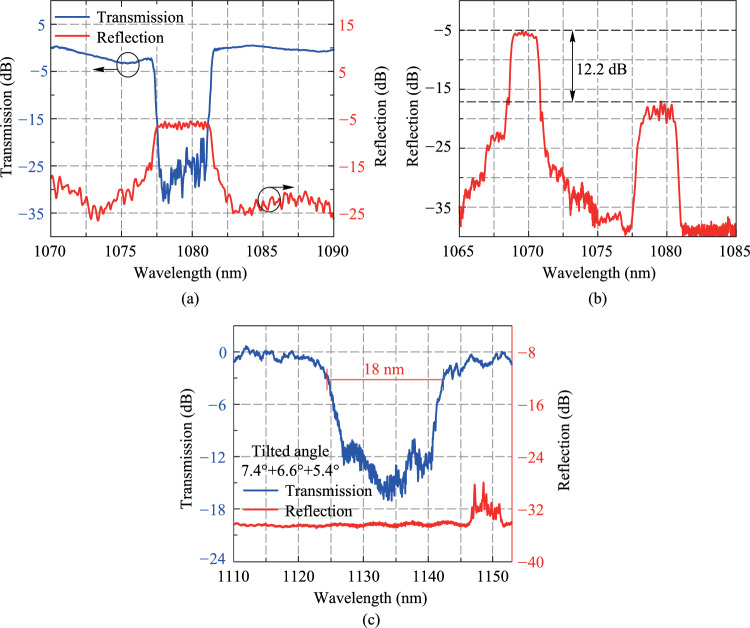
Measured spectra of **a** HRFBG, **b** LRFBG and **c** CTBFG

The high-reflectivity FBG (HRFBG) was inscribed in a 30 μm/600 μm LMA-DCF, and its spectrum was measured by the conventional FBG spectrum measurement system, as shown in Fig. 4a. We can see there are fluctuations of more than 2 dB in the transmission spectrum, which stem from two aspects. One is the fluctuation caused by the multimode fiber measurement system itself: The high-order modes in the measurement system cannot be completely filtered out by the MFA, causing fluctuations. On the other hand, the transmission spectrum of HRFBG may have cladding mode coupling loss in the short-wavelength direction, which may thus cause fluctuations in the transmission spectrum. It should be emphasized that, since the transmission spectrum depth of HRFBG is greater than 20 dB, which is far greater than the fluctuations in the transmission spectrum, the impact of fluctuations on reading the transmission spectrum depth of HRFBG can be neglected, and the corresponding reflectivity of HRFBG is greater than 99%. In the reflection spectrum of HRFBG, its central wavelength is 1079.3 nm and the 3-dB bandwidth is 4 nm. The LRFBG was inscribed on a 30 μm/250 μm fiber, and the reason for the reduced cladding size of LRFBG is to match the size of the output fiber of the pump combiner in the fiber oscillator system. The multimode LRFBG spectrum measurement system with scale FBG was used to measure its reflection spectrum, as shown in Fig. 4b. The scale FBG is an HRFBG, whose spectral characteristics are also measured via the conventional FBG spectrum measurement system. The central wavelength of the scale FBG is 1070 nm, with a transmission spectrum depth of 40 dB, corresponding to a reflectivity of 99.99%. In the reflection spectrum, the reflection peak intensity difference between the scale FBG and that of the multimode LRFBG is 12.2 dB, and the reflectivity of the LRFBG is 6% calculated from Eq. (1). The reflection spectrum shows that the center wavelength of multimode LRFBG is 1079.4 nm, and the 3-dB bandwidth is 2.1 nm. Subsequently, a CTFBG was inscribed on one side of the LRFBG to form a composite FBG, which can make the oscillator system more compact and stable. A cascaded inscription method is used to inscribe a cascaded CTFBG with different tilt angles [15], and the total transmission spectrum of cascaded CTFBG is equal to the superposition of the spectra of multiple CTFBGs, which can effectively enhance the bandwidth of the transmission spectrum. Through simulation design combined with actual inscription results after multiple iterative optimizations, three CTFBGs with tilted angles of 7.4°, 6.6°, and 5.4° are inscribed in cascade, with a total length of 40 mm. The spectrum of CTFBG is obtained by using the conventional multimode FBG spectrum measurement system, as shown in Fig. 4c. The central wavelength of the CTFBG transmission spectrum is 1135 nm with a 3-dB bandwidth of 18 nm, which corresponds to the first-order Raman wavelength of 1080 nm signal light.

## Application of FBGs in high-power lasers

**Fig. 5:**
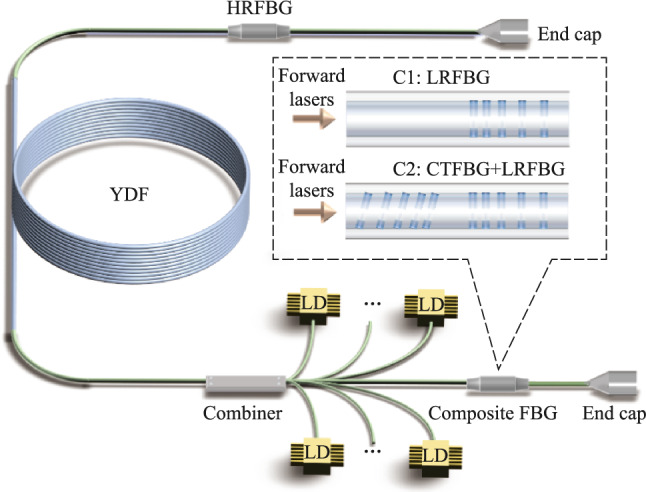
Schematic diagram of the high-power fiber oscillator using fs-written composite FBGs

Figure 5 presents the schematic diagram of the high-power fiber oscillator using fs-written composite FBGs. The fiber oscillator adopts the pure backward pumping scheme, and the gain fiber is a 30 μm/600 μm ytterbium-doped fiber (YDF) with a length of 38 m. The absorption coefficient of YDF at 915 nm wavelength is 0.37 dB/m, and the numerical aperture (NA) of the core is 0.058. A (36 + 1) × 1 backward pump/signal fiber combiner is used to inject pump light, and the input and output fibers of the fiber combiner are 30 μm/600 μm and 30 μm/250 μm LMA-DCF, respectively. The pump source adopts 969 nm + 982 nm dual-wavelength laser diodes (LDs), which are conductive to improve the threshold of transverse mode instability (TMI) [38, 39]. Neither the forward nor backward output ends are connected to the cladding light stripper (CLS), and the laser is output directly through the end cap. There are two main reasons for not using CLS at the output ends. On the one hand, because the fiber oscillator adopts the pure backward pumping scheme, there is no residual pump light at the forward output end. In addition, the optical–optical conversion efficiency of the fiber oscillator is relatively high, so there is less residual pump light at the backward output end. On the other hand, when no CLS is used at the forward output end, the output power of the fiber oscillator can continue to increase after reaching the TMI threshold. After the occurrence of TMI, the proportion of high-order modes signal light increases in the fiber core, resulting in an increase in signal light leaking from the fiber core to the cladding due to the bending and winding of YDF. If the signal light leaked to the cladding is filtered out by CLS, it will not only reduce the efficiency of the oscillator and affect the increase of output power but also cause the CLS to heat up rapidly due to the filtering of a large amount of cladding light, resulting in damage risk. Although the beam quality of the fiber oscillator degrades without CLS, the output power of the fiber oscillator can be further improved. The purpose of applying composite FBG in a high-power fiber oscillator is not only to verify its performance of SRS suppression but also the performance of power handling capability, which means that it is necessary to improve the output power of the fiber oscillator as much as possible. Moreover, since the signal lasers distributed in the cladding mode and the core mode transmit the composite FBG, the absence of CLS does not affect the verification of the power handling capability of the composite FBG.

It should be noted that the power scaling process of the fiber oscillator is performed in two phases. When only LRFBG is inscribed on the 30 μm/250 μm LMA-DCF, the output power of the fiber oscillator is increased to the maximum, i.e., configuration 1 (C1). Then, the LRFBG is removed from the fiber oscillator, and the CTFBG is inscribed on one side of the LRFBG to form the composite FBG. The composite FBG is introduced into the fiber oscillator and the CTFBG is located in the resonator cavity, i.e., configuration 2 (C2). This is because compared with CTFBG located outside the resonant cavity, CTFBG has a better SRS suppression effect in the resonant cavity [36]. Finally, the output power of the fiber oscillator increases to the maximum for the second time.

**Fig. 6:**
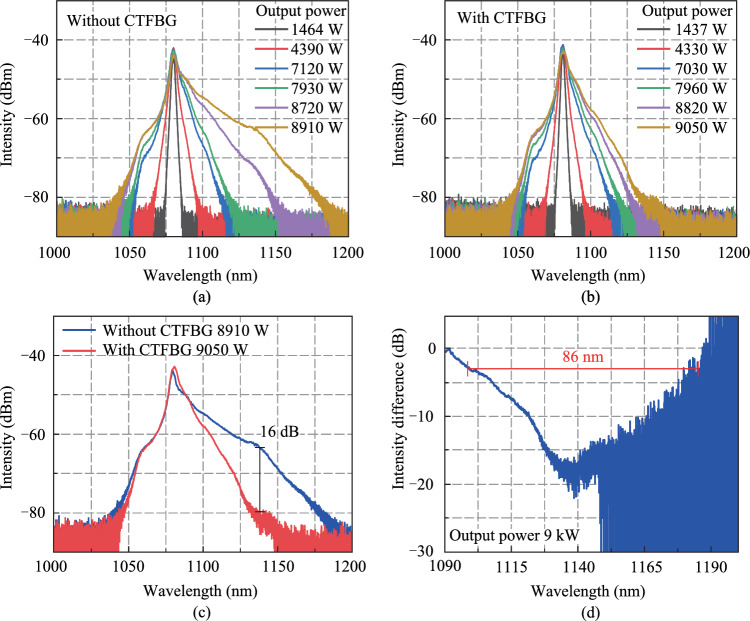
Output spectra at different output powers **a** without CTFBG and **b** with CTFBG. **c** Comparison of output spectra at the maximum output power without and with CTFBG. **d** SRS suppression spectrum of CTFBG at the maximum output power

Figures 6a and b present output spectra of the fiber oscillator at different output powers without CTFBG and with CTFBG, respectively. In configuration 1, Raman components are observed at 1135 nm waveband and SRS caused the spectrum to broaden rapidly when the output power is 8.7 kW. When the output power increases to 8.9 kW, the spectrum is broadened to nearly 1200 nm. In configuration 2, the output power can be further increased to 9 kW, and the SRS and spectral broadening are effectively suppressed. To better compare the changes in the output spectra, Fig. 6c shows the output spectra at the maximum output power without and with CTFBG. The CTFBG suppression spectrum obtained by subtracting two output spectra at the maximum output power is shown in Fig. 6d. It can be seen that the SRS suppression effect of CTFBG is very significant. The suppression spectral depth reaches 16 dB (97.5%), and the 3-dB bandwidth of the suppression spectrum reaches 86 nm.

**Fig. 7:**
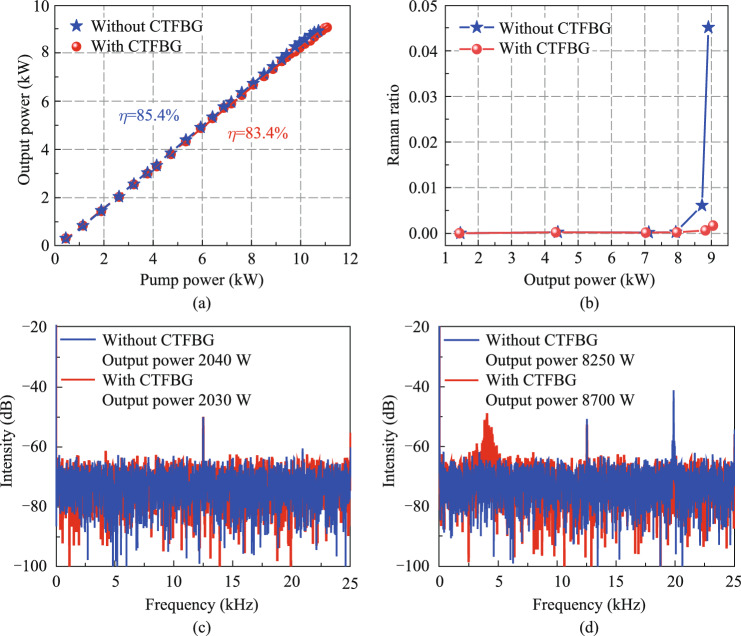
**a** Variation of output power with pump power without CTFBG and with CTFBG. **b** Variation of Raman ratio with output power without CTFBG and with CTFBG. The frequency spectrum corresponding to the output time domain signal when the TMI threshold is **c** not reached or **d** reached

Figure 7a presents the variation of the output power of the fiber oscillator with pump power without and with CTFBG, and the output power includes both cladding mode and core mode power. In configuration 1 without CTFBG, the maximum output power of the fiber oscillator is 8910 W, corresponding to the slope efficiency is 85.4%. The output power is limited by TMI. In fact, it can be seen from Fig. 7d below that TMI has already appeared at 8250 W, which could cause some light of the core mode to leak into the cladding mode. Since there is no CLS to filter the cladding light, the output power and efficiency are not decreased due to TMI. In configuration 2 with CTFBG, the slope efficiency decreases to 83.4% because the CTFBG partially filters out some Raman light and has insertion loss. Although the slope efficiency of the fiber oscillator decreases, the output power of the fiber oscillator is further increased to 9050 W due to the suppression of SRS and the increase of the TMI threshold. Similarly, power growth is still limited by TMI. The temperatures of CTFBG, HRFBG, and LRFBG with powerful cooling packages are measured by a thermal camera, and the temperatures increase linearly with the increase of signal power handled by the FBGs. At maximum output power, the temperature of three FBGs are all less than 50 °C. In addition, the Raman light ratio (the power ratio between the wavelength of 1120 nm and 1200 nm in the output spectrum) at different output powers is calculated through the output spectrum, as shown in Fig. 7b. Before the inscription of CTFBG, the Raman light ratio increases significantly when the output power increases to 8720 W, so the SRS threshold can be considered as 8720 W. As the output power further increases, the Raman light ratio increases exponentially, and the Raman light ratio at the maximum output power increases to 4.5%. After the CTFBG is inscribed to form a composite FBG, the Raman light ratio decreases from 4.5% to 0.2% at the maximum output power of 9050 W, indicating that the Raman light ratio in the output power decreases by an order of magnitude. Therefore, the CTFBG not only increases the output power from 8910 to 9050 W (with an increase of 140 W), but also improves the signal purity in output power. It is worth noting that CTFBG also raises the TMI threshold.

Figure 7c shows the frequency spectrum corresponding to the output time domain signal of the fiber oscillator when the output power is only 2 kW and lower than the TMI threshold. There is a frequency peak at 12 kHz, which is the basal frequency noise caused by the electrical signal. Figure 7d presents the frequency spectrum when the output power reaches the TMI threshold. As the output power increases, other frequency peaks appear in the frequency spectrum, indicating that the TMI occurs in the fiber oscillator. In configuration 1 without CTFBG, when the output power increases to 8250 W, the frequency peak at 20 kHz appears, indicating the occurrence of TMI. TMI could cause some light of the core mode to leak into the cladding mode, so it can be considered that 8250 W is the maximum power of the core mode. It should be emphasized that when power continues to increase after TMI occurs, the power leaking from the core mode to the cladding mode will increase. When the cladding mode power is too high, it may cause damage to the LD pump source through the pump combiner. Thus, continuing to increase power after TMI occurs carries certain risks. In configuration 2 with CTFBG, the TMI threshold is increased to 8700 W, and the corresponding frequency peak is 4 kHz. Since the frequency of intensity fluctuations caused by TMI is related to the power of the fiber oscillator, and the TMI threshold power increases after introducing CTFBG, the corresponding fluctuation frequency also changes. Therefore, the maximum power of the core mode also increases with the increase of the TMI threshold. Because SRS can induce the occurrence of TMI in fiber lasers, the TMI threshold can be increased when SRS is suppressed [40, 41]. Signal light is converted into Raman light via SRS, generating more heat in the fiber due to quantum defects. TMI is usually caused by thermal effects, so the heat generated by SRS can induce the occurrence of TMI.

The reported research results on suppressing SRS in fiber oscillators using CTFBG are shown in Table 1. The CTFBGs used in most of the works were inscribed by UV lasers. In this work, we used the CTFBG inscribed by fs lasers to suppress SRS in the fiber oscillator, achieving a record power level, which fully demonstrated that the fs-written CTFBG have greater potential for suppressing SRS in fiber oscillators. Moreover, we avoided increasing the number of splice points after the introduction of CTFBG by fabricating composite FBG, effectively reducing the impact of CTFBG on the compactness and stability of the oscillator system.

**Table 1:** SRS suppression in fiber oscillators by CTFBG

Fiber types	Inscription method	Number of CTFBGs	Number of splice points increased	CTFBG position	Output power/kW	Ref.
20/400 DCF	UV laser	1	2	Out of cavity	0.9	[11]
20/400 DCF	UV laser	1	2	In cavity	1.8	[13]
20/400 DCF	UV laser	1	1	Out of cavity	1.5	[42]
10/130 DCF	UV laser	1	1	In cavity	0.1	[43]
20/400 DCF	UV laser	2	2	In cavity	2	[14]
20/400 DCF	fs laser	1	1	In cavity	3.5	[36]
30/250 DCF	fs laser	1	0	In cavity	9	This work

## Conclusion

We demonstrate a composite FBG that can effectively suppress SRS in a high-power fiber oscillator and enhance the compactness and stability of the system. The composite FBG consists of a 1135 nm CTFBG and a 1080 nm LRFBG, both of which are fabricated in the same 30 μm/250 μm fiber using the fs-laser phase mask technology. By using the composite FBG, SRS is effectively suppressed with a Raman suppression depth and width of 16 dB and 86 nm respectively, and the maximum output power of the fiber oscillator is increased by 140 W to 9 kW, corresponding to the slope efficiency of 83.4%. The research results are expected to enhance the performance of high-power fiber oscillators and also to promote their use in a wider range of high-end manufacturing fields. The next work is to realize a fiber oscillator with an output power of more than 10 kW based on the fs-written composite FBG through comprehensively suppressing nonlinear effects such as SRS and TMI.

## Data Availability

Data will be made available on request.
